# The role of parental stress on emotional and behavioral problems in offspring: a systematic review with meta-analysis

**DOI:** 10.1016/j.jped.2024.02.003

**Published:** 2024-04-15

**Authors:** Larissa H. Ribas, Bruno B. Montezano, Maria Nieves, Luiza B. Kampmann, Karen Jansen

**Affiliations:** a Universidade Católica de Pelotas, Pelotas, RS, Brazil; b Universidade Federal do Rio Grande do Sul, Porto Alegre, RS, Brazil

**Keywords:** Parental stress, School-age children, Emotional problems, Internalizing problems, Behavior problems, Externalizing problems

## Abstract

**Objective:**

Empirical evidence underscores an association between parental stress and emotional and behavioral problems in offspring. However, a comprehensive systematic review or meta-analysis on this topic is lacking. Thus, this study aims to address the scientific inquiry: Is there a relationship between parental stress and emotional/behavioral problems in children?

**Sources:**

This systematic review with a meta-analysis surveyed PubMed, PsycINFO, and the Biblioteca Virtual em Saúde between August and September 2021. The present search combined terms (school-age children) AND (parental stress OR parenting stress OR family stress) AND (emotional and behavioral problems OR internalizing and externalizing problems). Eligibility criteria encompassed cross-sectional, cohort, and case-control studies published within the last five years, exploring the association between parental stress (stressful life events and parenthood-related stress disorders) and emotional/behavioral problems in school-age children. PROSPERO ID CRD42022274034.

**Summary of the findings:**

Of the 24 studies meeting all inclusion criteria (*n* = 31,183) for the systematic review, nine were eligible for inclusion in the meta-analysis. The meta-analysis revealed an association between parental stress and emotional problems (COR: 0.46 [95 % CI: 0.27 - 0.61], *p* < 0.001, Heterogeneity = 89 %) as well as behavioral problems (COR: 0.37 [95 % CI: 0.27 - 0.46], *p* < 0.001, Heterogeneity = 76 %).

**Conclusions:**

These findings indicate that parental stress predicts emotional/behavioral problems in school-age children. Since these problems are related to long-term negative effects in adulthood, these results are crucial for preventing mental health problems in offspring and for screening and managing parental stress.

## Introduction

Children with emotional and behavioral problems are at risk of long-term negative effects in adulthood. Children's emotional symptoms include anxiety, depressive symptoms, and withdrawal; therefore they are more likely to develop depressive disorders in the future.^1^ Furthermore, child-externalizing behaviors including inattention, opposition, hyperactivity, and aggression are associated with relationship and parenting difficulties, educational achievement, and substance abuse.^1^^,^^2^ Epidemiological studies have reported that the worldwide prevalence of mental disorders affecting children and adolescents was 13 %;^3^ specifically, up to 7 % and 9 % of children in the United States met the criteria for emotional and behavioral-related disorders, respectively.^4^

Epigenetics reveals that there are critical periods in childhood in which lived experiences can sculpt brain development.^5^ One of these moments is school age, a crucial period for the development of mental health problems^6^ due to the new socio-emotional and behavioral challenges that are now being experienced.^6^^,^^7^ Therefore, identifying the factors that influence early emotional and behavioral symptoms in school-aged children is important for preventing mental disorders in the future.^8^ Stressful environments have been found to exacerbate youth depression and anxiety during adolescence and may be a risk factor for the development of emotional and behavioral problems in children.^8^ While stress itself is not a pathology, chronic exposure to it increases the risk of psychopathology.^9^ Psychological distress in parents is particularly concerning, as it is associated with negative child and family outcomes, including youth adjustment problems and ineffective parenting.^10^

In particular, parenting stress - stress experienced by parents or caregivers related to their parenting role - has been identified as an important factor. Empirical evidence has consistently shown that parental stress is associated with emotional and behavioral problems in children.^1^^,^^2^^,^^9^, ^10^, ^11^, ^12^, ^13^, ^14^, ^15^, ^16^, ^17^, ^18^, ^19^, ^20^, ^21^, ^22^, ^23^, ^24^, ^25^ Attachment theory suggests that children establish an emotional and physical bond with their primary caregivers,^26^ with adults appearing to act as external regulators for children's emotional and behavioral functions.^20^ Given the lack of recent meta-analyses, this systematic review aims to clarify the significance of addressing both parents' mental well-being and their children's mental health. Thus, the authors aim to address the following question: Is there a relationship between parental stress and emotional/behavioral problems in children?

## Material and methods

The Preferred Reporting Items for Systematic Reviews and Meta-analysis (PRISMA) guidelines were adhered to for this review (http://prisma-statement.org/).

### Protocol registration

A protocol for this systematic review was prospectively registered in the International Prospective Register of Systematic Reviews (PROSPERO) under the ID “CRD42022274034” on June 20, 2022.

### Search strategy

The data sources employed in this study encompassed PubMed, *Biblioteca Virtual em Saúde* (BVS), and PsycINFO. The article search period spanned from August 2021 to September 2021. The present search strategy entailed a combination of the following terms: (school-age children) AND (parental stress OR parenting stress OR family stress) AND (emotional and behavioral problems OR internalizing and externalizing problems). Articles published in the last five years carried out in humans and without language restrictions, were included. Articles satisfying the inclusion criteria were those published within the past five years, conducted on human subjects, and without language restrictions. A total of 2.245 articles were retrieved (PubMed = 1.345, PsycINFO = 565, and BVS = 335), with 2.099 remaining after eliminating duplicates.

To determine whether an article was relevant to this study, the authors used the following inclusion criteria: (1) the study included school-age children (5 to 10 years old) and even studies that are not only conducted on school-age children, but include some of these ages, were included; (2) the study should link parental stress with child mental health; (3) the study included only fathers, only mothers, or both; (4) the study could include reports from children, parents, and/or teachers; and (5) the study should address parental stress, including stressful life events and parental stress disorders related to parenting. The exclusion criteria were: (1) studies that assessed the parents’ mental health, without including a specific measure of parental stress; (2) studies that only included biological measures of stress, not including self-reports of stress; (3) studies in which the children's pathology was a characteristic of the population and not the outcome; and (4) studies about specific populations were the stress caused by this specificity-like gender-expansive or children with organic diseases such as craniofacial anomalies.

The included study types were: 1) cross-sectional studies, and 2) longitudinal studies (cohort and case-control). Exclusion criteria were as follows: 1) systematic reviews, 2) other types of reviews, 3) case reports, 4) descriptive studies, and 5) meta-analyses.

In the present study, parental stress was employed as the exposure variable. The primary outcomes were childhood emotional and behavioral problems, assessed through reports from the children themselves, parents and/or caregivers, and teachers.

The studies were evaluated by two blinded raters, who determined their adherence to the inclusion criteria. The raters independently assessed the manuscripts using the Rayyan platform, resolving discrepancies through consensus among all authors. Initially, articles were screened based on title and abstract, followed by a full-text review. Articles not meeting the search criteria were excluded.

### Data extraction

Two researchers were engaged in the data extraction process. The authors collected information such as authors, year of publication, study location, study objectives, design, sample characteristics, assessments, and key findings pertaining to the correlation between parental stress and children's emotional and behavioral problems.

### Quality assessment

Each manuscript was independently evaluated by two blinded researchers using the Newcastle-Ottawa Quality Assessment Scale (NOQAS). Any disagreements were resolved through consensus among all authors.

### Statistical analysis

The authors conducted a descriptive synthesis of the findings (extracting author names, sample size, instruments, measure of effect, study aim, and other information listed in Item 26 of the form). To summarize the results of the selected articles, a meta-analysis was performed. The authors calculated the random effects estimates for meta-analyses with correlations of parental stress and (a) externalizing and (b) internalizing problems separately using inverse variance weighting for pooling. I2 (I2) was used to measure statistical heterogeneity. It is defined as the percentage of variability in effect sizes that is not caused by sampling errors. The analysis was performed using R programming language (version 4.2.2) with the meta package (version 6.0).

## Results

### Study selection

**Figure 1:**
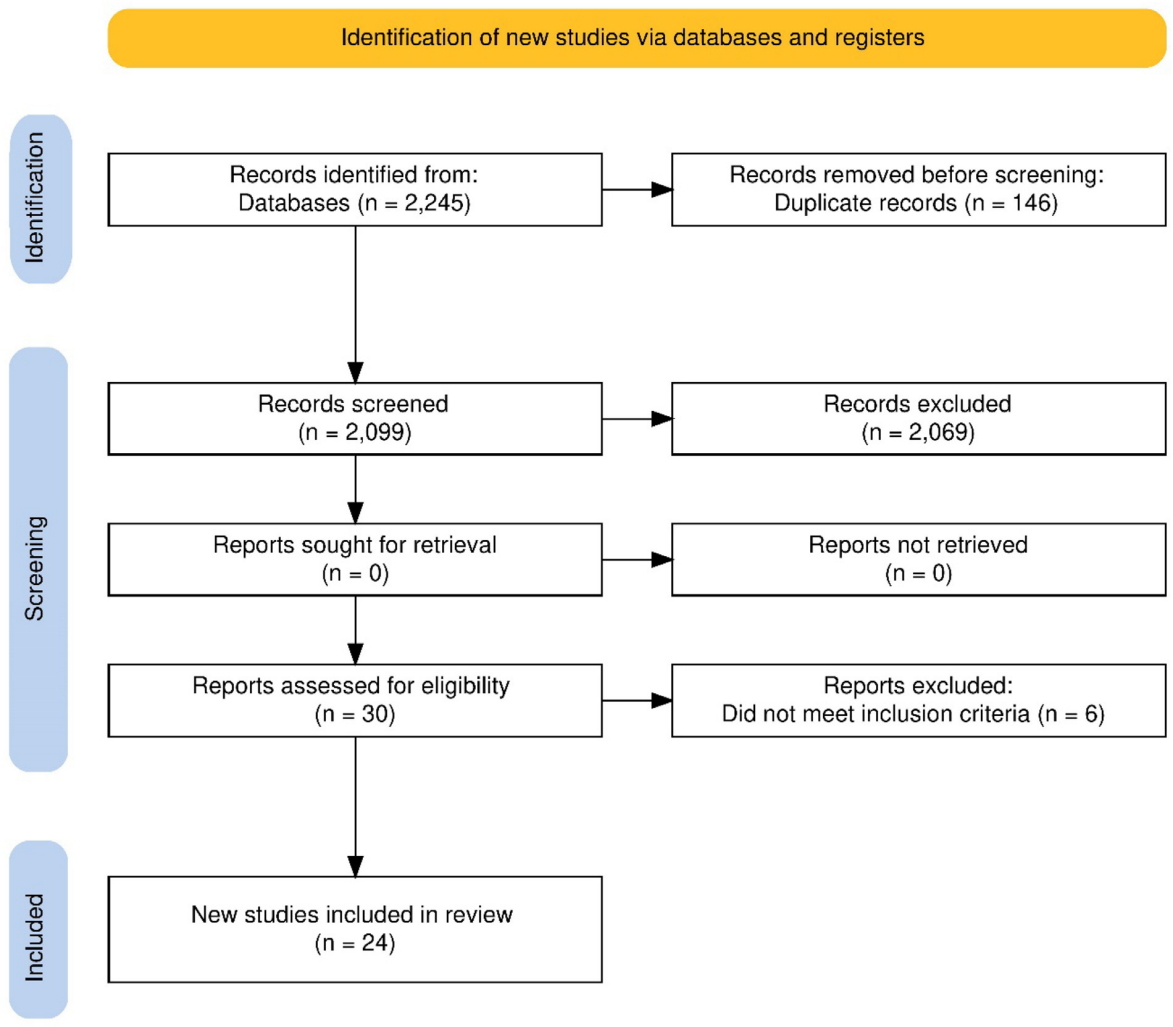
Flow diagram.

The literature search yielded 2.245 studies. Among these, 146 were duplicates, resulting in 2.099 potentially eligible studies, of which titles and abstracts were reviewed. At this stage, 2.074 studies did not meet the inclusion criteria, leaving 30 studies for full-text assessment. Ultimately, 24 studies met all inclusion criteria and were incorporated into the systematic review (Figure 1). The average quality score of the studies in the NOQAS was 7.16. Furthermore, the authors manually searched the references of the included studies and found no additional relevant studies.

### Study characteristics

Of the 24 included studies, 12 were cross-sectional ^1^^,^^2^^,^^9^, ^10^, ^11^, ^12^, ^13^, ^14^, ^15^, ^16^, ^17^^,^^27^,and 12 were longitudinal ^8^^,^^18^, ^19^, ^20^, ^21^, ^22^, ^23^, ^24^, ^25^^,^^28^, ^29^, ^30^ (*n* = 31.183). Parental stress was assessed using different instruments in the selected studies. The Parental Stress Index-Short Form was the most commonly used by the selected studies,^2^^,^^11^, ^12^, ^13^, ^14^^,^^17^^,^^20^^,^^22^^,^^27^^,^^28^^,^^30^ followed by the Perceived Stress Scale ^15^^,^^18^^,^^23^^,^^25^. In addition, several instruments have been used to assess emotional and behavioral problems in children. The most commonly used were the Child Behavior Checklist (CBCL),^2^^,^^10^, ^11^, ^12^^,^^15^^,^^16^^,^^18^^,^^21^^,^^28^, ^29^, ^30^ and the Strengths and Difficulties Questionnaire (SDQ) ^17^^,^^19^. The additional details of the selected studies are presented in Table 1.

**Table 1:** The main findings of the studies included in the systematic review.

Authors, year	Country	Objective	Study design	Sample characteristics	Parents' stress assessment instruments	Offsprings' diagnosis assessment instruments	Main outcomes	Is there an association between parental stress and emotional and behavioral problems of offspring?	Is this study eligible for meta-analysis?	Study quality (NOQAS)
Arbel et al., 2020	USA	To test how deviations in a mother's parenting stress (PS) levels across her child's transition to adolescence contribute to subsequent changes in her child's symptom levels	Longitudinal	202 mother-child dyads (community sample). Children's age: 8–12 years old (51.0 % female).	Parental Stress Scale	Revised Children's Anxiety and Depression Scale	Maternal attunement predicted reduced symptoms in children and lower maternal parental stress (PS) across waves 1–5. However, an inverse relationship between children's symptoms in wave 5 and maternal PS in wave 6 emerged. Children's baseline age is inversely related to PS in waves 1, 2, and 4, as well as with their symptoms in waves 1 and 2. A U-shaped pattern was observed in the concurrent link between mothers' PS and children's self-reported internalizing symptoms. The prospective association between maternal PS and children's symptoms was not significant	Yes	No	Eight
Chardon et al., 2016	USA	To examine the moderating role of youth sleep disturbance on the relationship between youth internalizing and externalizing symptoms and parent psychological distress	Cross-sectional	225 youths (outpatient sample). Youths's age: 8–17 years old (54.7 % female).	Brief Symptoms Inventory–18	Child Behavior Checklist	Greater internalizing symptoms, externalizing symptoms, and sleep disturbance in youth were found to correlate with increased parent psychological distress	Yes	No	Eight
Davis et al., 2017	Georgia	To study how preschoolers’ genetic, physiological and behavioral (i.e., negative emotionality) sensitivity factors interact with parenting stress to impact maternal perceptions of child adjustment across three domains: internalizing, externalizing, and sleep problems	Cross-sectional	108 dyads (community sample). Mean age: 3.50 years (61 % male).	Parenting stress index-short form	Emotion Regulation Checklist; Child Behavior Checklist	Child genetic sensitivity moderated the associations between parenting stress and child internalizing and sleep problems. Specifically, maternal parenting stress was significantly and positively associated with child sleep and internalizing problems, but only for children who exhibited high genetic sensitivity. Additionally, children's negative emotionality moderated the link between maternal parenting stress and child internalizing and externalizing problems, aligning with the principles of a diathesis-stress model	Yes	Yes	Seven
de Vries et al., 2017	The Netherlands	To examine the association of separate father-reported family adversity factors assessed pre and postnatally, in relation to children's bullying behaviors in early elementary school	Longitudinal	1298 children (community sample). Mean age: 7.53 years old (667 female).	General Functioning Scale of the McMasters Family Assessment Device	PEERS measure	Father-reported family adversity (ie. family distress) predicted children's bullying behaviors over and above the background family risk factors, early childhood externalizing problems and mother-reported family adversity. The association of fathers’ prenatal hostility and family distress with subsequent bullying behavior of their child at school was partly mediated by fathers’ harsh disciplinary practices at preschool age	Yes	No	Eigtht
Dubois-Comtois et al., 2021	Canada	To evaluate whether fathers’ levels of symptomatology and parenting stress were related to internalizing and externalizing behavior problems in preschool-aged children and whether quality of father–child interactions mediated this relation	Cross-sectional	81 two-parent families (community sample). Mean age: 48.36 months (53 % male).	Brief Symptom Inventory; Parenting Stress Index-Short	Achenbach System of Empirically Based Assessment	Fathers' and mothers' distress were associated with internalizing and externalizing problems	Yes	Yes	Seven
Gissandaner et al., 2020	USA	To investigate the extent to which caregiver's stress associated with relationships/responsibilities (RR), having basic needs and health concerns served as pathways between caregiver's victimization history and child's behavior outcomes	Cross-sectional	1.354 adult caregivers (community sample). Children's age: 4, 6, 8, 10, and 12 years old (*n* = 697 female).	Everyday Stressors Index	Child Behavior Checklist	Caregiver's everyday stress related to RR served as a mediator between caregiver's victimization history and increased children's internalizing symptoms. Caregiver's child victimization and combined victimization, but not adult victimization, was robustly related to baseline increases in child's internalizing symptoms; any caregiver victimization history significantly predicts RR stress; and compared to basic needs and health/environmental concerns, RR stress was identified as a robust mediator between caregiver's victimization and children's baseline internalizing symptoms	Yes	No	Six
Hentges et al., 2019	Canada	To test alternative theories about the underlying mechanisms behind the association of maternal prenatal stress and child psychopathology	Longitudinal	1992 mother–child pairs (community sample). Children's age: 5 years old	Perceived Stress Scale	Child Behavior Checklist	Prenatal stress continued to exert a direct effect on internalizing problems at age five, even after controlling for postnatal stress, birthweight, hostile-reactive parenting, and child's negative affect. However, prenatal stress was only indirectly related to child's behavior problems at age five, through multiple pathways, including postnatal stress, hostile parenting, and child's negative affect	Yes	No	Eight
Hosokawa, Katsura, 2021	Japan	To clarify the relationship between parents’ work–life balance (WLB) and children's mental health, as well as the underlying factors of parental stress and nurturing attitude	Cross-sectional	473 youths and caregivers (community sample). Youths age: 10–11 years old (52.2 % female)	Perceived Stress Scale	Strengths and Difficulties Questionnaire	Even after adjusting for children's gender, family composition, family income, and parental educational attainment, it was observed that the higher the work–family negative spillover, the higher the child's externalizing and internalizing problems. The results indicated that maternal WLB was related to children's behavior both negatively and positively through the paths of maternal stress and parenting practices	Yes	No	Eight
Kolbuck et al., 2019	USA	To describe the relations between psychological functioning, parenting stress, and parental support in clinicreferred, prepubertal gender-expansive children and to examine parental support and parenting stress as moderators of the relationship between children's gender nonconformity and psychological functioning	Cross-sectional	71 youths (community sample). Child's age: 3–11 years old (70 % male at birth).	The Parenting Stress Inventory—Short Form	The Child Symptom Inventory and the Early Childhood Inventory	Parenting stress significantly predicted higher symptom counts across all 8 diagnoses. Parenting stress was a significant moderator of relations between gender nonconformity and attention-deficit/hyperactivity disorder hyperactive–impulsive type and conduct disorder symptoms; higher levels of gender nonconformity were associated with higher symptom counts as moderate and high levels of parenting stress (but not at low levels of parenting stress)	Yes	No	Five
Liu et al., 2018	China	To examine the reciprocal relations between 3 dimensions of parenting stress (i.e., Parental Distress, Parent–Child Dysfunctional Interaction, and Difficult Child) and their children's Oppositional Defiant Disorder (ODD)	Longitudinal	Initially, 243 dyads (community sample). Children's ages: 6–12 years old (72.8 % male)	Parenting Stress Index–Short Form, numérica	Eight-item ODD diagnostic scale in DSM–IV	Parent–Child Dysfunctional Interaction (PCDI) positively predicted children's ODD symptoms; ODD symptoms positively predicted parental perceptions of Difficult Child and PCDI. Children's ODD symptoms significantly exacerbated parenting stress in Difficult Children and Parent–Child Dysfunctional Interaction, which in turn was associated with higher Parental Distress. Further, children's ODD symptoms positively predicted all 3 dimensions of parenting stress at T3	Yes	No	Seven
Lohaus et al., 2018	Germany	To investigate the longitudinal relationships between foster children's mental health problems and parental stress	Longitudinal	94 foster children and 157 biological children (community sample). Children's age: 2–7 years old in both sample	Parental Stress Questionnaire	Child Behavior Checklist	Associations between children's mental health problems and parental stress were in general higher for externalizing in comparison to internalizing problems. Increases (or decreases) in children's symptoms were related to corresponding increases (or decreases) in parental stress. Changes in externalizing symptoms were related to changes in stress perceptions in mothers and fathers of both samples, while changes in internalizing symptoms were related to changes in maternal stress only in foster families	Yes	No	Eight
McDaniel and Radesky, 2018	USA	To investigate longitudinal bidirectional associations between parent's technology use and child's behavior, and understand whether this is mediated by parenting stress	Longitudinal	337 parents (community sample). Children's age: 0–5 years old (55 % female)	Parenting Stress Index	Child Behavioral Checklist	Child behavioral difficulties – especially externalizing -were associated with later higher levels of parent stress, which in turn were associated with higher downstream technology use during parent-child activities	Yes	No	Eight
Neppl et al., 2016	USA	To understand how economic hardship is associated with externalizing problems in young children. Specifically, parental emotional distress, observed couple conflict, and observed hostile parenting were assessed when the child was between the ages of three and five years old	Longitudinal	451 families (community sample). Children's age: 2, between 3 and 5, and 6 to 10 years old (*n* = 236 females)	Emotional distress	Child Behavior Checklist	Economic pressure, emotional distress, and couple conflict are associated with parenting and thus may contribute to externalizing problems in later childhood. Parental emotional distress was also significantly correlated with couple's conflict, harsh parenting and externalizing behaviors in children between the ages of 6 and 10	Yes	Yes	Seven
Parent et al., 2021	Canada	To relate the parents' perceived stress and the children's internalizing and externalizing problems and if clustering pro-inflammatory cytokines by their profile levels in saliva can predict the emotional function of children aged 0–17 in response to caregiver perceived stress	Cross-sectional	622 child-caregiver dyads (outpatient sample). Children's age: 7 years old (52 % female)	Perceived Stress Scale	Child Behavior Checklist	Cytokine clusters did significantly moderate the association between increased caregiver perceived stress and reduced child emotional functioning and increased Attention-Deficit-Hyperactivity problems. Using a cytokine clustering technique may be useful in identifying those children exposed to increased caregiver perceived stress that are at risk of emotional and attention deficit hyperactivity problems	Yes	No	Six
Parkes and Sweeting, 2018	Scotland	To explore how mothers’ perceptions of social and formal support when children were ages 10 –22 months predicted trajectories of children's externalizing and internalizing problems from 58 to 122 months	Longitudinal	3.031 families were followed to the final time point (community sample). Children's age: 70, 94, and 122 months	Depression, Anxiety, and Stress Scale; Short Form Health Survey; Parental Stress Scale	Strengths and Difficulties Questionnaire	Social support reduced effects of economic strain on internalizing problems, and formal support reduced effects of dysfunctional parenting on internalizing problems	Yes	Yes	Eight
Samuelson et al., 2016	USA	To examine if parenting stress and maternal emotional availability would mediate relationships between maternal posttraumatic stress disorder and children's emotional and behavioral functioning	Cross-sectional	52 mothers-children (community sample). Children's age: 7–12 years old (57 % male)	Parenting Stress Index Short Form	Emotion Regulation Checklist; the Child Behavior Checklist	Parenting stress was strongly related to all three child functioning variables (emotion regulation, internalizing, and externalizing behaviors)	Yes	Yes	Five
Simons, Cillessen e Weerth, 2016	The Netherlands	To investigate whether cortisol stress responses of 6-year-olds were associated with their behavioral functioning at school	Cross-sectional	149 children (community sample). Mean age: 6.09 years old (*n* = 70 girls)	Parenting Stress Index	Teacher Report Form and Preschool Social Behavior Questionnaire	Children of mothers with more parenting stress were seen as less prosocial by their teacher. Although these findings do not indicate the absence of the moderating role per se, they may suggest that family stress does not have a general effect on the association between cortisol stress responses and behavioral functioning	No	Yes	Nine
Tokunaga et al., 2019	Japan	To investigate the relationship between the parenting stress experienced by parents of non-clinical preschool children and the children's behavioral characteristics	Cross-sectional	83 pairs of mothers and fathers (community sample). Mean age: 59.1 months (*n* = 47 female).	Parenting Stress Index–Short Form	Strengths and Difficulties Questionnaire	Parenting stress experienced by fathers was significantly related to hyperactivity/inattention, while parenting stress experienced by mothers was significantly related to peer relationship problems and emotional symptoms	Yes	No	Eight
Tuovinen et al., 2020	Finland	To examine if maternal antenatal symptoms of depression, anxiety and perceived stress were associated with mental and behavioral disorders in their children, if the associations varied according to gestational week, stress type, fluctuating or consistently high symptoms, and if they were driven by maternal or paternal lifetime mood or anxiety disorders	Longitudinal	3365 women (community sample). Children's age: 6–10 years old (51.6 % male)	Perceived Stress Scale	International Statistical Classification of Diseases and Related Health Problems-10 (ICD-10)	The hazard of any childhood mental and behavioral disorder was significantly higher for children whose mothers reported consistently high in comparison to consistently low levels of all types of stress throughout pregnancy. Maternal antenatal stress is associated with higher risk of childhood mental and behavioral disorders	Yes	No	Seven
van Eldik et al., 2017	the Netherlands	To examined dynamic associations between marital stress and children's externalizing behavior	Longitudinal	369 two-parent families (community sample). Mean age: 7.70 years (53,9 % girls)	Parenting Stress Index	Child Behavior Checklist	The main results support the idea of codevelopment between marital stress and externalizing behavior	Yes	Yes	Seven
van Vugt et al., 2015	USA	To identify possible family and parenting variables that may help explain the increased risk for future persistent delinquent behaviour of children born to mothers who were younger than average	Longitudinal	247 youths (community sample). Youth's age: 7 to 19 years (all male participants)	Perceived Stress Scale	Child Behavior Checklist	Parents who consistently experience stress may be more likely to lash out at their children in anger and have difficulties effectively managing the competing demands associated with maintaining a household. A chaotic and even hostile family environment may, in turn, negatively impact on the emotional functioning of the child, leading to development of disruptive and delinquent behaviors	Yes	Yes	Seven
Vidal et al., 2016	Chile	To analyze the role of parenting stress as a variable that mediates the relationship between socio-economic status (SES) and both externalized and internalized behaviors in preschool children	Cross-sectional	16.033 children and their caregivers (community sample). Mean age: 4.5 years old (51 % girls)	Parenting Stress Index	Child Behavior Checklist	This study suggests that the relationship between SES and externalized and internalized behaviors of preschool children would be mediated by the level of family stress, especially parental stress	Yes	No	Seven
Whitson and Kaufman, 2017	USA	To relate whether parental stress influences children's exposed to potentially traumatic events outcomes in the health care system	Longitudinal	184 parents/caregivers (outpatient sample). Children's age: 1–5 years old (75.0 % male)	The Parenting Stress Index - Short Form	Child Behaviour Checklist	The results indicated that the families enrolled in this early childhood system of care evidenced significant reductions in parenting stress and child internalizing and externalizing behaviors	Yes	No	Seven
Wu et al., 2018	USA	To test a model of parenting stress as a mediator between maternal depressive symptoms, emotion regulation, and child behavior problems	Cross-sectional	119 mothers-child dyad (community sample). Children's age: 0–6 years old (*n* = 66 boys)	Parenting Stress Inventory Short Form	Child Behaviour Checklist	Maternal parenting stress was associated with elevated child externalizing and internalizing problems. Maternal depressive symptoms were positively associated with externalizing problems	Yes	Yes	Six

Parenting stress (PS); Relationships/responsibilities (RR); Oppositional Defiant Disorder (ODD); Parent–Child Dysfunctional Interaction (PCDI); Posttraumatic stress disorder (PTSD); Socio-economic status (SES).

### Studies included in the meta-analysis

**Figure 2:**
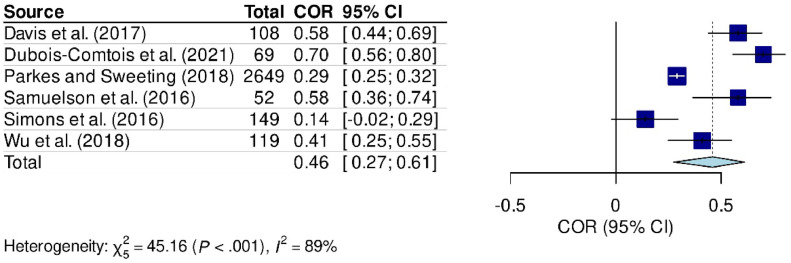
Meta-analysis comparing the relationship between parental stress and emotional problems in school-aged children.

**Figure 3:**
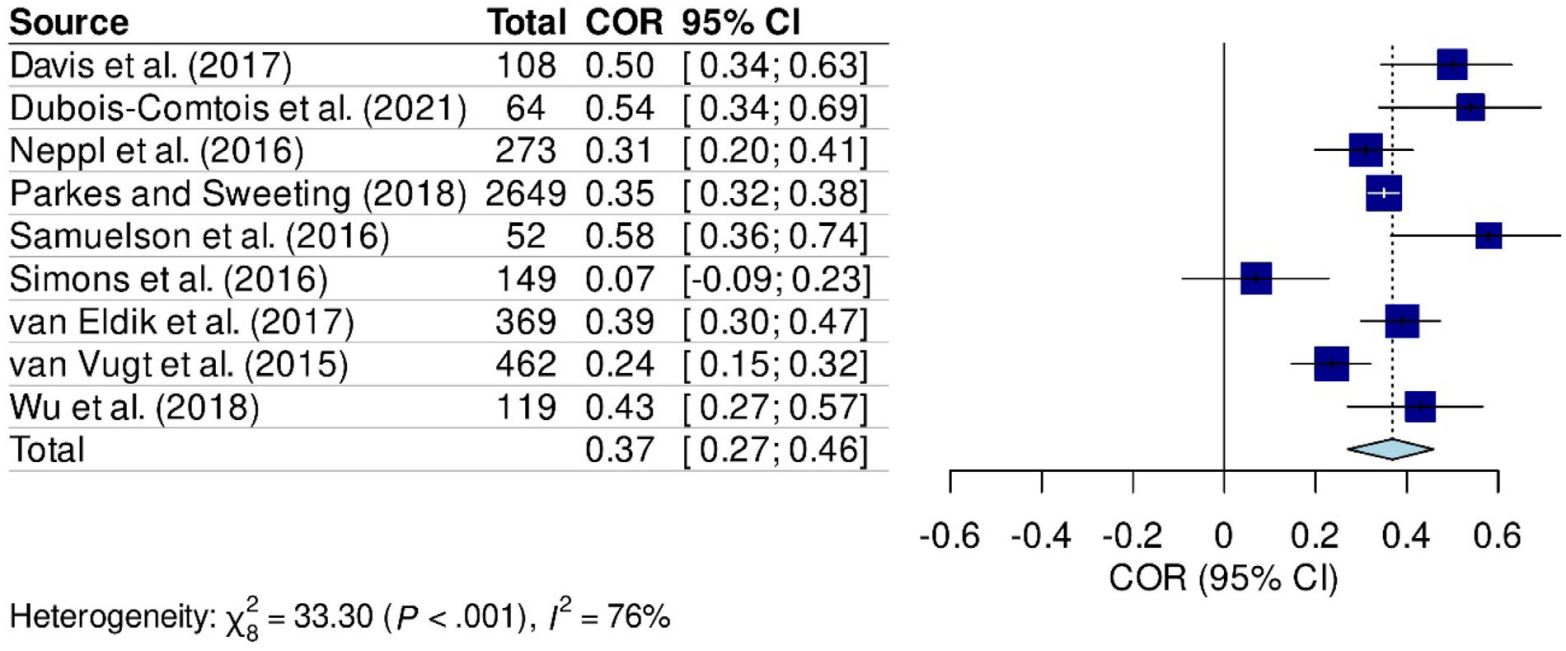
Meta-analysis comparing the relationship between parental stress and behavioral problems in school-aged children.

Of these studies, nine had sufficient data to be included in the meta-analysis.^2^^,^^12^^,^^14^^,^^16^^,^^18^^,^^19^^,^^21^^,^^27^^,^^28^ In the model for emotional problems, six studies were included (Figure 2), whereas in the model for behavioral problems, nine studies were included (Figure 3).

## Results related to parental stress and emotional and behavioral problems in the offspring

### Evidence from cross-sectional studies

Parental stress is significantly associated with emotional and behavioral problems in offspring. ^1^^,^^2^^,^^9^, ^10^, ^11^, ^12^, ^13^, ^14^, ^15^, ^16^, ^17^ Additionally, there was variation in the issues manifested by offspring based on the caregiver's stress experience. Notably, paternal parenting stress showed a significant association with hyperactivity/inattention, while maternal parenting stress correlated significantly with peer relationship problems and emotional symptoms. ^17^ Parental stress has further been linked to childhood sleep disorders ^10^ and the regulation of children's emotional function.^12^ A study unveiled the correlation between maternal parental stress, child sleep, and emotional problems solely in younger children with high genetic sensitivity.^2^ Parental stress also acts as a mediator between maternal post-traumatic stress disorder and offspring's emotional regulation, as well as emotional and behavioral problems.^12^ Moreover, it serves as a significant moderator for the relationship between gender nonconformity and ADHD hyperactive-impulsive type and CD symptoms.^13^ Nonetheless, no significant associations were discovered between maternal parenting stress and children's emotional and behavioral problems in a particular study.^27^

### Evidence from longitudinal studies

Several studies have explored parental stress as a potential exposure, with children's emotional and behavioral problems as subsequent outcomes. Parents who consistently experience stress may be more likely to lash out at their children in anger and have difficulties effectively managing the competing demands associated with maintaining a household. A chaotic and even hostile family environment may, in turn, negatively impact the emotional functioning of the child, leading to the development of disruptive and delinquent behaviors,^18^ which are behavioral problems.

In addition, social and formal support effects mediated mainly via lower maternal distress were associated with lower child emotional and behavioral problem trajectories via lower dysfunctional parenting.^19^ Moreover, parenting stress mediated the relationship between the number of potentially traumatic events a child experienced and potentially mediated traumatic events and emotional problem behaviors.^20^ In addition, parental emotional distress was significantly correlated with couples’ conflict, harsh parenting, and externalizing behaviors in children.^21^ There were dynamic relations between parenting stress, parent–child interaction, and children's ODD.^22^

Prenatal parenting stress has also been associated with emotional and behavioral problems during childhood.^23^ Father-reported family adversity, which includes prenatal family stress, predicts children's bullying behaviors.^24^ Children whose mothers reported consistently high levels of all types of stress during pregnancy were at a higher risk of emotional and behavioral problems.^25^

On the other hand, other studies have presented children's problems as predictors of parental stress. One study demonstrated that even though emotional and behavioral scores were substantially correlated with parental stress, there was no clear pattern of temporal relationships between children's mental health scores and parental stress.^29^ Results from other research suggested a two-way dynamic in which parents, stressed by their child's difficult behavior, may withdraw from parent-child interactions through technology use, and this could influence child externalizing and withdrawal behaviors in the offspring over time.^30^ In addition, mothers' parental stress contributes to their children's emotional trajectories which may vary as a function of deviations in maternal attunement.^8^

Finally, one study showed a reciprocal relationship between marital stress and perceived parental competence over time. Two elicitation effects appeared during adolescence, showing that parents who reported higher behavioral problems in early adolescence reported more marital stress and a lower sense of competence later.^28^

### Meta-analysis results

The meta-analysis results confirmed an association between parental stress and emotional problems in school-age children (COR: 0.46 [95 % CI: 0.27 - 0.61], *p* < 0.001, Heterogeneity = 89 %) (Figure 2), as well as behavioral problems (COR: 0.37 [95 % CI: 0.27 - 0.46], *p* < 0.001, Heterogeneity = 76 %) (Figure 3). Sensitivity analysis was not conducted due to the quality of studies assessed using the NOQAS.

## Discussion

The majority of the selected studies found correlations between parental stress and childhood outcomes. Twelve cross-sectional studies and twelve longitudinal studies were included. Among the cross-sectional studies, eleven confirmed the correlation between parental stress and emotional or behavioral problems, and one rejected this hypothesis. Among the longitudinal studies, eight looked at parental stress as exposure and at children's emotional and behavioral problems as outcomes, three presented children's problems as predictors of parents’ stress, and one focused on the co-development between marital stress and behavioral problems in the offspring. Meta-analyses confirmed the relationship between parental stress and emotional and behavioral problems in school-age children.

To date, in the last 5 years, no systematic review has addressed the question: Is there a relationship between parental stress and emotional and behavioral problems in the offspring? The systematic review^31^ that is most similar to ours, also assessed maternal mental health, but through the short-and long-term effects of prenatal exposure to untreated maternal depressive symptoms - and not parental stress itself. The study showed that depressive-like conduct is more frequently detectable in newborns whose mothers experienced depressive symptoms during pregnancy than in neonates born to healthy mothers or mothers diagnosed with depression at the onset of puerperium.^31^ In addition, newborns of mothers with prenatal symptoms of depression may more frequently exhibit a disposition toward behavioral inhibition and negative affectivity. Notably, this study evaluated the effects of antenatal exposure to untreated maternal depressive symptoms, and the aim of the present study was to evaluate the effect of maternal stress on the development of emotional and behavioral problems in the offspring.^31^

Similar to depression, parental stress can also be experienced during the prenatal period. Exposure to prenatal parental stress is related to the later development of emotional and behavioral problems in offspring.^19^^,^^23^^,^^25^ Importantly, parental responsibilities, which can contribute to parental stress, may even be perceived by parents before the child is born. Consequently, conducting new research to assess parental stress during the prenatal period could prove crucial for the early identification and prevention of such stress, even before the child's birth. This has the potential to create an environment conducive to better emotional health right from the outset of the child's life. The insights from this research could be invaluable for healthcare professionals, enabling them to address the issue during prenatal pediatric consultations and obstetric appointments, thereby fostering interdisciplinary support. Encouraging the establishment of support groups focused on promoting parental mental health, led by an interdisciplinary team of obstetricians, pediatricians, psychologists, and psychiatrists, could prove beneficial.

It's important to emphasize that, in order to care, caregivers need to be cared for. Care involves managing one's stress levels through healthy relationships, nutritious meals, adequate sleep, physical activity, mindfulness, and caring for one's own mental health.^32^ So, it's essential that healthcare professionals, including Pediatricians, are aware of these factors involved in caregiving, to direct attention and efforts towards promoting caregiver care. Recognizing and early preventing parental stress, as well as providing support to parents in managing stressful events related to parenthood, is one way of taking care of those who care, and also of encouraging caregivers' self-care.

The parents are essential to support and assist in the development of basic social and emotional skills, which can allow children to be resilient, despite the adversities they may face. Attachment theory suggests that children are predisposed to form a strong emotional and physical attachment to at least one primary caregiver.^26^ Thus, the caregiver's substantial support is essential for the basis of children's emotional, behavioral, and social functioning,^33^ acting as an “external regulator” in the socialization of children's emotions through the instruction, modeling, and definition of behavioral expectations.^20^ Situations that may impair parental mental health, such as parental stress, can ultimately hinder parental support and assistance for basic child skills, reflecting in the mental health of the offspring, as the authors observed through the present findings.

The authors all need to embrace and spread the concept of relational health, that is, a child's ability to develop and maintain safe, stable, and nurturing relationships with others.^34^ Caregivers should actively promote positive relational experiences throughout childhood,^34^ with themselves as well as with the people who live with the child. In this sense, care for the caregiver is also important, as it was observed that not only is there a relationship between parental stress and emotional and behavioral problems in the offspring, but parental stress can also lead to some parental practices and parental conflicts that can harm the health of the relationships. For example, parental stress can also provide chaotic,^18^ hostile environments,^18^ couples conflict,^21^ severe parenting,^18^ and remove parents from interactions with their children through the use of technology,^30^ which can also be harmful to the establishment of healthy relationships.

In addition, identified emotional problems related to parental stress include GAD,^13^ MDD,^13^ dysthymia,^13^ social anxiety disorder,^13^ and the regulation of children's emotional function.^12^ The behavioral problems evidenced included ADHD,^13^^,^^17^ ODD,^13^^,^^22^ CD^13^ and disruptive and delinquent behaviors.^18^ In addition, parental stress is also related to sleep disorders,^2^^,^^10^ and problems with peers^17^ and is a significant moderator of the relationship between gender nonconformity and ADHD hyperactive-impulsive type and CD symptoms.^13^

In view of the above, early recognition and prevention of parental stress are important in preventing emotional and behavioral problems in the offspring. Since emotional/behavioral problems can lead to impairments not only in childhood but also in the long term,^1^^,^^2^ identifying factors associated with these problems is a crucial step in preventing potential mental health issues in both childhood and adulthood. In this regard, Pediatricians play a significant role as they can follow families from prenatal pediatric consultations and the child's birth, thereby enabling early identification of signs and symptoms of parental stress and suggesting interdisciplinary support with psychological/psychiatric assistance. In addition, early diagnosis and prevention practices can be positive for both parents, individually and as couples, as well as for their children. These practices may be helpful in promoting physical and mental health by improving children's sleep disorders, parenting, and relationships for school-age children, and providing less dysfunctional environments.

During childhood, there are sensitive and critical periods during which lived experiences can sculpt brain development, as revealed by epigenetics.^5^ School-aged is one such moment. This period of life is crucial for the development of mental health problems,^6^ as it is the moment when new interactions with teachers and classmates begin, and when difficulties in fulfilling expectations become more noticeable for the child.^6^ In addition, children face more challenges such as peer pressure, acceptance, and labeling.^7^ The results of the meta-analysis confirmed the relationship between parental stress and emotional and behavioral problems in school-age children. Therefore, research on mental health conducted with school-age children can provide important information for the development of preventive practices for emotional and behavioral problems, and also how to identify them.

The limitations of this study include the heterogeneity of parental stress evaluation measures. It was included here mostly in studies that evaluated stress with The Parental Stress Index-Short Form and the Perceived Stress Scale, but other measures were used. Emotional and behavioral measures were assessed mostly using SDQ and CBCL. However, in not every study, the respondents were the same (sometimes the mother, sometimes the father), and there was variability in the interpretation of the results. The findings must be taken with caution due to the range of age included in this study - it was selected studies where the children were between five and ten years of age; therefore, these findings cannot be used in other age groups. Further research should be conducted to amplify this age range. Furthermore, systematic reviews are subject to publication bias, as it is easier to publish studies that have confirmed the relationship between exposure and outcome than studies with non-significant results.

To conclude, parental stress is related to emotional and behavioral problems in the offspring and could also be a predictor of those outcomes in school-aged children. This study may serve as a guide to the development of public health policies that should focus on childhood mental health prevention, with early intervention in childhood and evaluation and intervention focused on parents’ mental health.

## Conflicts of interest

The authors declare no conflicts of interest
